# Impact of the COVID-19 Pandemic on Routine Childhood Vaccination in Oklahoma

**DOI:** 10.3390/vaccines14030271

**Published:** 2026-03-19

**Authors:** Jessica Beetch, Laura A. Beebe, Amanda Janitz, Chao Xu, Mary Gowin, Katrin Gaardbo Kuhn

**Affiliations:** 1 Medical School Program in Health Disparities Research and School of Public Health, University of Minnesota, Minneapolis, MN 55414, USA; 2 Department of Biostatistics and Epidemiology, University of Oklahoma Health Sciences, Oklahoma City, OK 73104, USA; laura-beebe@ouhsc.edu (L.A.B.); amanda-janitz@ouhsc.edu (A.J.); chao-xu@ouhsc.edu (C.X.); katrin-kuhn@ouhsc.edu (K.G.K.); 3 Department of Health Promotion Sciences, University of Oklahoma Health Sciences, Oklahoma City, OK 73104, USA; mary-gowin@ou.edu

**Keywords:** COVID-19, pandemic, vaccination, Oklahoma, DTaP, MMR, children

## Abstract

**Background/Objectives**: The COVID-19 pandemic substantially disrupted routine childhood vaccination practices across the United States. Oklahoma, a state characterized by lower socioeconomic indicators and higher levels of vaccine hesitancy, may have been particularly vulnerable to these disruptions. This study aimed to assess the impact of the COVID-19 pandemic on routine childhood vaccination in Oklahoma. **Methods**: Data were obtained from the Oklahoma State Immunization Information System to examine changes in the administration of DTaP and MMR vaccines before and during the COVID-19 pandemic, stratified by pandemic phase. Percentage changes in vaccine doses administered were calculated across time periods. Log-binomial regression models were used to evaluate the association between pandemic timing and receipt of subsequent DTaP doses among children under one year of age. **Results**: Administration of both DTaP and MMR vaccines declined during the COVID-19 pandemic across all pandemic phases examined. Compared with the pre-pandemic period, fewer children returned for subsequent DTaP doses during the pandemic. Regression analyses indicated a reduced likelihood of completing age-appropriate DTaP dosing among infants during the pandemic. **Conclusions**: Routine childhood vaccination in Oklahoma declined during the COVID-19 pandemic, with persistent reductions observed across pandemic phases. These findings highlight vulnerabilities in vaccination delivery during public health emergencies and underscore the need for targeted state-level strategies to sustain routine immunization services during future crises.

## 1. Introduction

The COVID-19 pandemic placed unprecedented strain on healthcare systems worldwide, leading to substantial disruptions in the delivery and utilization of routine health services [1]. Routine vaccination was one of the most affected services, with widespread declines in vaccination coverage reported across the United States [2]. An analysis of 10 jurisdictions in the United States found routine vaccinations were 15.7% to 71.3% lower in March to May 2020 compared with the same periods in 2018 and 2019, with variations by vaccine type, age, and location [3]. Similar declines in routine vaccination during the pandemic have been documented in individual states, including Louisiana [4], Michigan [5], and Colorado [6].

Diphtheria, tetanus, and pertussis (DTaP) and measles, mumps, and rubella (MMR) are two common routine childhood vaccines administered throughout the United States. DTaP is administered as a five-dose series across early childhood, while MMR is administered in two doses during early childhood and school entry [7]. Together, these vaccines have reduced mortality from diphtheria, tetanus, pertussis, measles, mumps, and rubella by nearly 100% in the United States [8]. Disruptions to routine childhood vaccination place children at risk for vaccine-preventable diseases associated with severe and lifelong consequences, including paralysis, hearing loss, and even death [9].

Understanding how routine childhood vaccination was affected during the COVID-19 pandemic is critical for strengthening health system preparedness and resilience during future public health emergencies. Evaluating pandemic-related disruptions in childhood vaccination can help identify system-level vulnerabilities, emerging health disparities, and opportunities to mitigate long-term consequences of missed or delayed vaccinations.

Oklahoma represents a particularly important setting for examining these disruptions. The state has a poverty rate above the national average, with 1 in 5 children living in poverty [10], and consistently ranks among the least vaccinated states in the country [11]. An assessment of state-level healthcare system performance during the COVID-19 pandemic ranked Oklahoma 50th worst nationally, only ahead of Mississippi (with inclusion of the District of Columbia) [12]. In Oklahoma, routine childhood vaccinations such as DTaP and MMR are required for attendance in childcare and school settings. In contrast to some countries or states where childhood vaccinations are compulsory with limited or no non-medical exemptions, Oklahoma allows exemption pathways for medical, religious, or personal reasons. Prior to the pandemic, exemption rates were comparable to the national average, with 2.6% of Oklahoma kindergarteners exempt from required vaccines in the 2018–19 school year compared with 2.5% nationally [13]. Exemption rates increased during the pandemic, reaching 4.7% in Oklahoma in the 2022–23 school year compared with 3.0% nationally [11]. Given these contextual factors, we hypothesized that the impact of the COVID-19 pandemic on routine childhood vaccination in Oklahoma may differ from patterns observed in other regions of the United States. The objective of this research study was to evaluate changes in childhood DTaP and MMR vaccination in Oklahoma by comparing routine vaccination during the COVID-19 pandemic with pre-pandemic levels.

## 2. Materials and Methods

We utilized vaccination data from the Oklahoma State Immunization Information System (OSIIS), a population-based registry to which all providers administering state-funded vaccines are required to report vaccinations for those 18 years of age and younger. While reporting of privately funded vaccinations is not mandated, many facilities voluntarily submit these records to OSIIS.

We examined DTaP and MMR vaccinations among individuals 18 years of age and younger in Oklahoma. These two vaccines were selected because they represent routinely administered childhood vaccinations with established schedules and high baseline uptake, making them useful indicators of broader trends in vaccine dose administration within the state. We additionally analyzed a sub-cohort of children <1 to 11 months of age receiving DTaP vaccines, as three of the five recommended DTaP doses are administered during the first year of life. Because MMR doses are administered years apart, we did not analyze the number of MMR doses received per child.

We conducted a retrospective cohort study combined with an ecological comparison of vaccine counts, designed to descriptively assess temporal trends in vaccination. Total DTaP and MMR doses administered during the COVID-19 pandemic (March 2020 to July 2022) were compared with a pre-pandemic baseline period (March 2017 to July 2019). Periods were matched by calendar month and duration to account for seasonal variation. Vaccine dose counts were also examined by pandemic phase, initial impact (March to September 2020), initial recovery (October 2020 to May 2021), Delta variant predominance (June to November 2021), and Omicron variant predominance (December 2021 to July 2022), and were compared to baseline counts.

For the infant sub-cohort, we assessed unique children under 1 year of age receiving either a single DTaP dose or more than one dose before versus during the pandemic to evaluate changes in routine vaccination uptake. The outcome variable was number of DTaP doses (single vs. more than one), and the primary exposure was time period (pre-pandemic vs. pandemic). Covariates evaluated as potential confounders or effect modifiers included gender, race, ethnicity, and community-level median household income. Covariates were selected a priori based on theoretical relevance and prior literature identifying determinants of vaccine uptake. A large portion of race and ethnicity data was missing (8.8% missing race data and 19.4% missing ethnicity), as has been observed across other populations during the pandemic [14]. Community-level median household income was categorized as $59,999 and below or $60,000 and above using statistics from the US Census Bureau. Reported estimates of Oklahoma’s state median household income in 2022 vary slightly across data sources, with one reporting $59,673 [15] and another reporting $61,364 [16]. Given this variability, we selected $60,000 as a cut point to distinguish areas with median household incomes above and below the approximate state median. We used ZIP Code tabulation areas from residential addresses to determine median household income for most ZIP Codes in the state. We substituted county median household income for the few areas without ZIP Code-level income data.

Percentage change was calculated for total DTaP and MMR vaccine dose count differences before and during the pandemic overall and by pandemic phase using the equation below.

$$\frac{\left(Count\;During-Count\;Before\right)}{Count\;Before}\times 100$$

Log-binomial regression was used to estimate risk ratios (RRs) and 95% confidence intervals (CIs) among infants, evaluating the association between time period and the binary outcome of single or multiple DTaP vaccine doses. Analyses were conducted in SAS version 9.4 (SAS Institute). A type 1 error rate of 0.05 was used to test for statistical significance.

## 3. Results

### 3.1. Characteristics of Included Children

The study included 627,663 children from birth to 18 years of age. Of these, 49.0% were girls and 51.0% were boys. Most were White (61.3%), followed by American Indian or Alaska Native (18.1%), Black or African American (11.0%), other race (6.3%), Asian (2.6%), and Native Hawaiian or Other Pacific Islander (0.7%). Just over 20.0% were Hispanic or Latino and 62.1% resided in areas with a median household income of $59,999 and below (Appendix A).

### 3.2. Percentage Change

**Figure 1:**
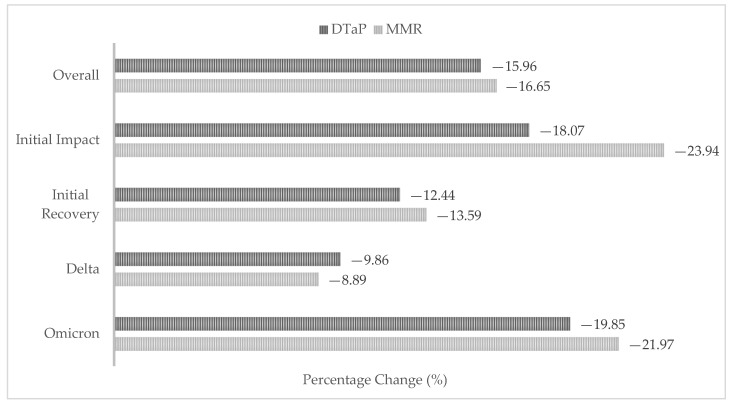
Percentage change in DTaP and MMR vaccine dose counts during COVID-19 pandemic phases for children 0–18 years of age.

A total of 1,999,836 DTaP and MMR vaccine doses were recorded on OSIIS for children from birth to 18 years of age during the overall study period. There was a 16.0% decrease in total DTaP vaccine doses administered and a 16.7% decrease in total MMR vaccine doses administered during the COVID-19 pandemic compared to before it began. Dose counts decreased during all four pandemic phases when compared to pre-pandemic counts (Figure 1). Dose counts before and during the pandemic can be found in the Appendix A.

### 3.3. Regression Analysis

A total of 213,900 unique children under 1 year of age who received at least one DTaP vaccination were included in the regression analysis (Table 1). Gender, race, ethnicity, and income were not identified as effect modifiers or confounders of the association between time period and DTaP vaccination dose. The risk of receiving more than one dose of DTaP vaccine was reduced by 7.6% (RR = 0.92, 95% CI: 0.92, 0.93) during the pandemic compared to before it began. This indicates that fewer infants returned for subsequent DTaP doses during the pandemic.

## 4. Discussion

In this large population-based descriptive study of children in Oklahoma, we observed substantial declines in routine childhood vaccination counts during the COVID-19 pandemic, along with evidence that fewer children returned for subsequent vaccine doses. These findings are consistent with prior research studies documenting widespread disruptions to routine childhood vaccination services across the United States during the pandemic. While some other studies reported similar findings, such as a 16% decline in DTaP vaccine administration and 22% decline in MMR vaccine administration in children under 2 years of age, others saw declines up to 62% in young children and 96% in older children [2]. Our results confirm that Oklahoma experienced declines in routine childhood vaccination similar to those observed in other regions. However, because Oklahoma has higher poverty rates and greater levels of vaccine hesitancy compared with many other states, disruptions to vaccination services may have more serious downstream implications. Declines in vaccination in already under-vaccinated settings could exacerbate existing disparities and increase the risk of vaccine-preventable disease outbreaks in the future.

Our study introduced pandemic phases defined by variant surges in Oklahoma. The largest declines in routine childhood vaccine dose administration were observed during the initial impact phase of the pandemic and again during Omicron variant predominance. These findings indicate that vaccine disruptions were not limited to the early months of the pandemic but occurred during multiple phases. Several factors may help explain these declines. During the initial impact phase, Oklahoma issued “Safer at Home” orders (commonly referred to as lockdown), which postponed minor medical procedures and closed non-critical businesses from 24 March to 6 May 2020 [17]. In addition, the COVID-19 pandemic altered health-seeking behavior, with many families delaying or avoiding healthcare visits due to concerns about infection risk [1]. It is plausible that these policies, along with reduced in-person healthcare utilization early in the pandemic, contributed to declines in routine childhood vaccination. Similarly, declines observed during Omicron variant predominance coincided with a surge in COVID-19-associated hospitalizations in January 2022 in the United States. Heightened healthcare system strain and the prioritization of COVID-19-related care during this time likely further disrupted routine vaccination services [18].

There was a higher proportion of American Indian or Alaska Native children recorded in OSIIS compared to state race demographics. This variation may be attributed to increased reporting from tribal facilities compared to private practices in the state. The lower proportion of White children and higher proportion of racially minoritized groups in OSIIS may reflect the socioeconomic makeup of facilities reporting vaccine data to OSIIS [16]. The number of children with missing race data more than tripled during the COVID-19 pandemic compared to before it began. Even before the pandemic, there was a substantial number of children with missing ethnicity data. Considering this, there could potentially be health disparities that remain unknown and warrant further investigation.

Several limitations should be acknowledged. First, only two routine childhood vaccines were studied. DTaP and MMR vaccines were selected because they are widely administered routine vaccines in the state. However, a decline in their administration would likely indicate similar trends for other routine vaccines. Second, the median household income data collected by ZIP Code may not precisely reflect the household income of each individual child included in the study. Third, we dichotomized some variables, including median household income and vaccine dose, to provide clear and easily interpretable assessments of trends. While this approach facilitates interpretation, more nuanced categorizations could provide additional insights. Fourth, race and ethnicity data were missing for a subset of the children in the dataset, which may influence interpretation of demographic patterns. We did not impute missing race or ethnicity data because imputing socially constructed variables could raise ethical concerns and introduce additional bias. Fifth, data entry was conducted by numerous individuals across thousands of facilities, which may have resulted in misclassification. Sixth, we did not account for population change. Though the birth rate has gradually declined in Oklahoma over the past decade, the state has experienced an uptick in its population of children since 2020. Despite this growth, fewer children received routine vaccinations during the pandemic, suggesting a downward trend in uptake. Our objective was to assess temporal trends in vaccine dose administration rather than precise coverage estimates. Finally, the descriptive nature of our analysis may not fully capture underlying temporal trends in vaccine dose administration. It is possible that trends present prior to March 2020 may not be fully distinguished from pandemic-related changes.

## 5. Conclusions

Our findings demonstrate that administration of both DTaP and MMR vaccinations declined during the COVID-19 pandemic compared to pre-pandemic levels in Oklahoma. These results highlight the vulnerability of routine childhood vaccination programs to largescale public health disruptions and underscore the need for proactive, resilient vaccination strategies. This evidence can inform guidance and preparedness planning to mitigate disruptions to vaccination services during future public health emergencies and to support timely recovery of vaccine administration.

## Figures and Tables

**Table 1:** Characteristics of children < 1 to 11 months of age that received a single vaccine dose or more than one vaccine dose of DTaP vaccine before and during the pandemic.

Characteristics	Before COVID-19		During COVID-19	
	1 March 2017–31 July 2019		1 March 2020–31 July 2022	
	Single Dose	More than One Dose	Single Dose	More than One Dose
	Count (%)			
Total	3843	109,222	11,475	89,360
Race				
American Indian or Alaska Native	330 (8.96)	19,232 (18.52)	1092 (17.46)	13,060 (21.56)
Asian	99 (2.69)	2654 (2.56)	186 (2.97)	1600 (2.64)
Black or African American	549 (14.91)	11,756 (11.32)	740 (11.83)	6839 (11.29)
Native Hawaiian or Other Pacific Islander	53 (1.44)	735 (0.71)	52 (0.83)	339 (0.56)
White	2240 (60.85)	57,684 (55.54)	3814 (60.97)	32,471 (53.60)
Other	410 (11.14)	11,800 (11.36)	372 (5.95)	6275 (10.36)
Ethnicity				
Hispanic or Latino	510 (15.20)	18,492 (19.00)	978 (15.17)	11,230 (18.71)
Not Hispanic or Latino	2846 (84.80)	78,840 (81.00)	5467 (84.83)	48,781 (81.29)
Median Household Income				
$59,999 and Below	2381 (63.04)	66,883 (62.38)	6652 (60.02)	53,222 (60.89)
$60,000 and Above	1396 (36.96)	40,332 (37.62)	4431 (39.98)	34,187 (39.11)

## Data Availability

The data that support the findings of the study are available from the Oklahoma State Immunization Information System. Raw data are not publicly available to preserve individuals’ privacy.

## Appendix A

**Table A1:** Characteristics of children 0–18 years of age receiving DTaP and/or MMR vaccines before COVID-19 emergence, during COVID-19 emergence, and total numbers using data from OSIIS.

Characteristics	Before COVID-19	During COVID-19	Total	Chi Sq *p*-Value
	1 March 2017– 31 July 2019	1 March 2020– 31 July 2022		
	Count (%)			
Gender				
Girl	197,699 (49.06)	110,136 (49.03)	307,835 (49.04)	0.7577
Boy	205,217 (50.93)	114,489 (50.96)	319,709 (50.95)	
Other	25 (0.01)	25 (0.01)	73 (0.01)	
Missing	28	21	49	
Race				
American Indian or Alaska Native	68,105 (17.46)	35,238 (19.37)	103,343 (18.07)	<0.0001
Asian	9823 (2.52)	4872 (2.68)	14,695 (2.57)	
Black or African American	42,560 (10.80)	20,052 (11.02)	62,612 (10.95)	
Native Hawaiian or Other Pacific Islander	3013 (0.77)	1200 (0.66)	4213 (0.74)	
White	242,777 (62.24)	108,087 (59.42)	350,864 (61.34)	
Other	23,785 (6.10)	12,463 (6.85)	36,248 (6.34)	
Missing	12,929	42,759	55,688	
Ethnicity				
Hispanic or Latino	71,161 (21.11)	33,963 (20.10)	105,124 (20.77)	<0.0001
Not Hispanic or Latino	266,015 (78.89)	135,041 (79.90)	401,056 (79.23)	
Missing	65,816	55,667	121,483	
Median Household Income				
$59,999 and Below	246,287 (62.28)	136,458 (61.87)	382,745 (62.13)	0.0013
$60,000 and Above	149,151 (37.72)	84,106 (38.13)	233,257 (37.87)	
Missing	7554	4107	11,661	

**Table A2:** Percentage change in vaccine dose counts before and during the COVID-19 pandemic for children 0–18 years of age.

Time	Dose Count Before COVID-19	Dose Count During COVID-19	Count Difference	Percentage Change
Overall	1 March 2017– 31 July 2019	1 March 2020– 31 July 2022		
DTaP	809,691	680,475	−129,216	−15.96%
MMR	277,971	231,699	−46,272	−16.65%
Initial Impact	1 March 2019– 30 September 2019	1 March 2020– 30 September 2020		
DTaP	209,817	171,900	−37,917	−18.07%
MMR	74,285	56,498	−17,787	−23.94%
Initial Recovery	1 October 2018– 31 May 2019	1 October 2020– 31 May 2021		
DTaP	191,571	167,748	−23,823	−12.44%
MMR	66,515	57,475	−9040	−13.59%
Delta Variant Predominance	1 June 2019– 30 November 2019	1 June 2021– 30 November 2021		
DTaP	174,195	157,025	−17,170	−9.86%
MMR	60,786	55,383	−5403	−8.89%
Omicron Variant Predominance	1 December 2018– 31 July 2019	1 December 2021– 31 July 2022		
DTaP	221,546	177,572	−43,974	−19.85%
MMR	77,527	60,492	−17,035	−21.97%
